# Low frequency of radiolucent lines in 3D‐printed porous‐coated tibial cementless baseplate at 1‐year follow‐up

**DOI:** 10.1002/jeo2.70356

**Published:** 2025-07-13

**Authors:** Yoshinori Mikashima, Hitoshi Imamura, Koichiro Yano, Katsunori Ikari, Hiroshi Takagi, Ken Okazaki

**Affiliations:** ^1^ Oume Knee Surgery Center, Takagi Hospital Ome Japan; ^2^ Department of Orthopaedics Tokyo Women's Medical University Shinjuku‐ku Japan; ^3^ Department of Orthopaedics Tokyo Women's Medical University Adachi Medical Center Adahi‐ku Japan

**Keywords:** 3D‐printed porous‐coated, cementless, radiolucent lines, total knee arthroplasty

## Abstract

**Purpose:**

A recently introduced 3D‐printed porous‐coated tibial baseplate (ATTUNE AFFIXIUM, DePuy Synthes) was hypothesised to yield radiographical and clinical results that are comparable to those of its cementless predecessor with sintered beaded porous coating (POROCOAT, DePuy Synthes).

**Methods:**

A consecutive series of total knee arthroplasties (TKAs) was performed by a single surgeon using a baseplate covered with either AFFIXIUM® 3D‐printed porous coating or POROCOAT® sintered beaded porous coating. The presence of radiolucent lines (RLLs) and bone on‐growth over the surface of the tibial baseplate were retrospectively reviewed at 1 week, 1, 2, 4, 6 months and 1 year postoperatively. The 2011 Knee Society Score, Forgotten Joint Score‐12 (FJS‐12), hip–knee–ankle (HKA) angle were also reviewed. One‐to‐one matching was performed for age, sex, body mass index and preoperative University of California, Los Angeles score. Fisher's exact test or independent Student's *t*‐test was used for statistical analyses.

**Results:**

Fifty AFFIXUM® and 50 POROCOAT® cementless TKAs were reviewed after 1:1 matching at 1 year postoperatively. RLLs appeared in 15 knees (30%) of POROCOAT® TKAs at 6 months postoperatively, whereas RLLs appeared in two knees (4%) of AFFIXIUM® TKAs (<0.01). RLLs in 6 of 15 POROCOAT TKAs (40%) and 1 of 2 AFFIXIUM TKAs (50%) disappeared 1 year postoperatively. Bone on‐growth over the surface of the tibial baseplate was observed in the same way in both groups. There were no significant differences in the 2011 Knee Society Scores and Forgotten Joint Scores‐12 between the cohorts, with no patients requiring revision surgery (not significant).

**Conclusion:**

A very low frequency of RLLs was observed on a newer 3D‐printed porous‐coated tibial baseplate design for TKA at 1‐year follow‐up.

**Level of Evidence:**

Level III, retrospective cohort study.

**Abbreviations**
+: positive
−: negative
a_hka: hip–knee–ankle after surgery
A: anterior
AP: anterior−posterior view
b_ext: extention before surgery
b_flx: flexion before surgery
b_hka: hip–knee–ankle before surgery
BMI: body mass index
bone ongr: bone on‐growth
ext: expectation score of 2011 Knee Society Score
FJS: Forgotten Joint Score
Fun: functional activity score of 2011 Knee Society Score
Gen: gender
k_sc: knee score
kl: Kellgren−Lawrence grade
L: lateral
Late: lateral view
M: medial
M: month
obj: objective knee score of 2011 Knee Society Score
P: posterior
RLL: radiolucent line
sat: satisfaction score of 2011 Knee Society Score
UCLA: UCLA score

## INTRODUCTION

Advances in manufacturing technology have allowed cementless implants to incorporate improved bioengineering designs, leading to better osteointegration [2, 4, 5, 10, 15]. As a result, several reports on modern cementless porous coatings have been described in the literature, including sintered titanium beads [3, 4], layered cobalt–chrome beads with peri‐apatite [5], 3D‐printed titanium [8], 3D‐printed tantalum [7] and porous plasma spray coating with hydroxyapatite [2]. These porous‐coated implants achieved excellent biological fixation and good mid‐to‐long clinical results [2, 3, 5, 10, 15].

3D‐printed porous titanium coating is thought to biologically resemble bone structure and elasticity and is therefore amenable to osteoblast colonisation and bone on‐growth [17]. A new 3D‐printed porous titanium coating, AFFIXIUM® (DePuy Synthes), was designed based on trigonal trapezohedron building blocks. The rhombus space is thought to be highly interconnected with bone on‐growth channels with evenly distributed pore diameters. Tong et al. reported superior bony ingrowth in AFFIXIUM® when compared with POROCOAT® (DePuy Synthes) in animal models [18]. However, to the best of our knowledge, there are few clinical and radiographical reports of this newer porous coating in cementless total knee arthroplasty (TKA).

The purpose of this retrospective study was to evaluate the short‐term radiographical and clinical results of a cementless TKA with a recently introduced 3D‐printed porous‐coated tibial baseplate, AFFIXIUM®. The newer cementless coating was hypothesised to yield results that are comparable in radiographical and clinical results to those of its cementless predecessor with sintered beaded porous coating (POROCOAT, DePuy Synthes) [3, 4].

## MATERIALS AND METHODS

**Figure 1:**
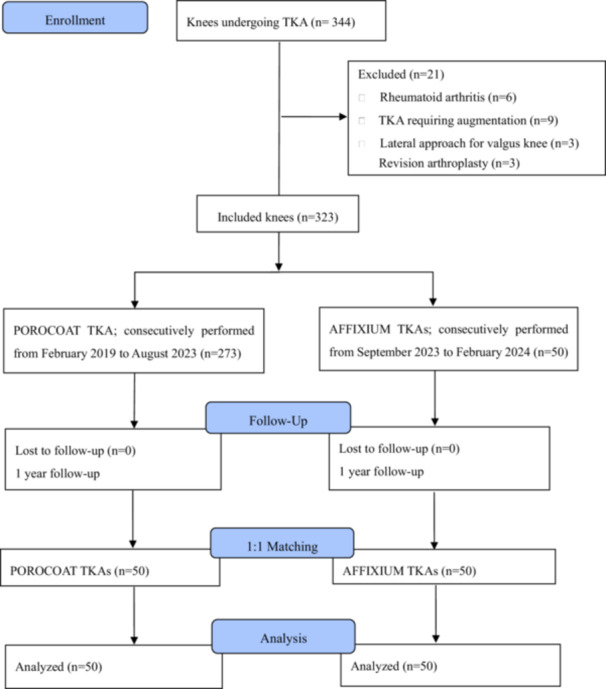
CONSORT flowchart of the patient inclusion process from enrolment to analysis. TKA, total knee arthroplasty.

A single‐institution retrospective study of prospectively collected data was performed. Patients who underwent primary TKA with AFFIXIUM (AFFIXIUM TKA) or TKA with POROCOAT (POROCOAT TKA) from February 2019 to February 2024 were enroled. POROCOAT TKAs were performed on consecutive patients between February 2019 and August 2023. Because AFFIXIUM TKAs were introduced to the Japanese market in September 2023, AFFIXIUM TKAs were performed on consecutive patients between September 2023 and February 2024. All procedures were performed with the approval of the institutional review board at our hospital in accordance with the ethical standards in the 1964 Declaration of Helsinki (approved number: 2025001). The inclusion criterion was defined as a diagnosis of Kellgren–Lawrence grade 4 primary osteoarthritis. The exclusion criteria were rheumatoid arthritis and revision arthroplasty. All patients were of Asian descent. Table 1 summarises the preoperative demographic information of the patients in the two groups. No patient was lost to follow‐up (Figure 1).

**Table 1:** Comparison of baseline demographics, preoperative clinical data and radiographic findings between the matched POROCOAT TKAs and AFFIXIUM TKAs.

	POROCOAT (*n *= 50)	AFFIXIUM (*n *= 50)	*p* value
Age (year)	73.0 ± 7.0	73.0 ± 7.7	0.10 NS
Sex	37 females	37 females	
	13 males	13 males	
BMI	26.7 ± 4.5	26.9 ± 4.3	0.35 NS
UCLA activity score (pt)	6.0 ± 0.3	6.0 ± 0.2	1.0 NS
Knee score (pt)	42.1 ± 16.2	39.8 ± 12.8	0.41 NS
Functional score (pt)	51.4 ± 12.5	48.2 ± 9.1	0.17 NS
HKA (d)	170.6 ± 5.8	171.0 ± 4.8	0.69 NS
Preoperative ROM (d)	109.0 ± 14.6	113.9 ± 14.4	0.09 NS

*Note*: Bold values indicate statistically significant.

Abbreviations: BMI, body mass index; d, degrees; HKA, hip–knee–ankle; NS, not significant; pt, points; ROM, range of motion; TKA, total knee arthroplasty; UCLA, University of California, Los Angeles.

All surgical procedures were performed by a single surgeon (Y.M.) via the medial parapatellar approach and capsulotomy with patellar eversion. The measured resection technique was adopted for femoral and tibial bone resections. Identical posterior‐stabilised (PS) mobile‐bearing polyethylene inserts were used for POROCOAT TKAs and PS fixed‐bearing polyethylene inserts were used for AFFIXIUM TKAs. Cemented patellar resurfacing via anatomically shaped patellar components was performed in both groups. The same femoral components were also used, surface treated with POROCOAT. AFFIXIUM TKAs have tibial baseplates with 3D‐printed titanium porous coating, whereas POROCOAT TKAs have sintered titanium bead coating. The AFFIXIUM TKAs feature a tibial baseplate with a central cruciform keel surrounded by four pegs to provide a press‐fit fixation, whereas the POROCOAT TKAs feature a central conical keel surrounded by four pegs to achieve press‐fit fixation.

**Figure 2:**
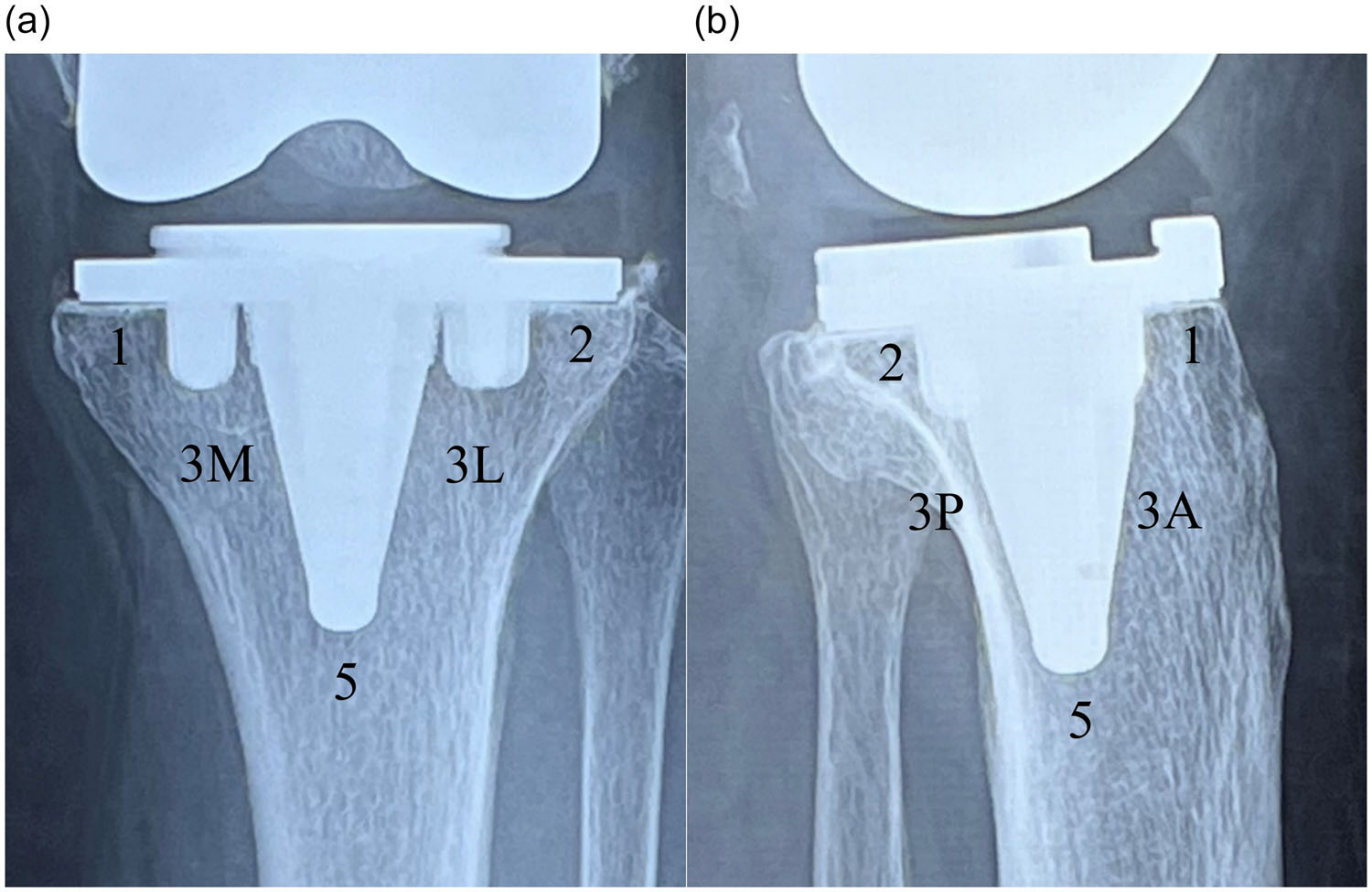
(a) Coronal radiographs of implants with zones for documentation of radiolucent lines. Zone 1: medial baseplate. Zone 2: lateral baseplate. Zone 3: central keel region. ‘M’ and ‘L’ are used to designate ‘medial’ and ‘lateral,’ respectively. Zone 5: the most inferior area of the keel. (b) Sagittal radiographs of implants with zones for documentation of radiolucent lines. Zone 1 of the tibia: anterior baseplate. Zone 2 of the tibia: posterior baseplate. Zone 3 of the tibial: central keel region. ‘A’ and ‘P’ are used to designate ‘anterior’ and ‘posterior,’ respectively. Zone 5 of the tibia: the most inferior area of the keel.

The primary investigative item of this study was the frequency of radiolucent lines (RLLs) and bone on‐growth to the tibial tray. In addition, the secondary investigative item was the clinical outcomes. The 1‐week, 1‐, 2‐, 4‐, 6‐month and 1‐year postoperative radiographs were obtained, reviewed and assessed to determine the occurrence of component subsidence or failure. Two serial radiographs were obtained by radiology technicians. If serial radiographs were not available, another radiograph was taken to obtain serial radiographs. RLLs were evaluated at the bone‐implant interface via the Modern Knee Society Radiographic Evaluation System [11]. Three general zones were defined on the anteroposterior (AP) and lateral views of the tibial implant: Zones 1 and 2 to designate the periphery, Zone 3 to indicate the central keel region and Zone 5 to denote the most inferior region of the keel (Figure 2a,b). Since the same femoral implants were used in both groups, zones of the femur were not investigated. Bone on‐growth over the surface of the tibial baseplate was defined as positive if bone on‐growth greater than 0.5 mm was observed in at least one zone. An RLL was defined as a visible radiolucency of 0.5 mm or greater, encompassing at least one zone. The radiographic image was enlarged, and a digital measurement tool (Synapse®; Fuji Medical) was used to measure the maximum width of the RLL. The results between the two groups were subsequently compared.

The measurement of the width of RLLs was performed by the author (Y.M.) with extensive experience in using the measurement system. The assessment was not blinded. The widths of the RLLs were also measured in 20 randomly selected radiographs to verify their intra‐ and interrater reliability. Measurements were performed twice by the same examiner with an interval of more than 2 months between measurements. Additionally, measurements were also performed by another examiner (H.I.) with extensive experience in using the measurement system. The interclass correlation coefficient (ICC) was assessed between groups.

Preoperative data collection included knee scores, functional scores, University of California, Los Angeles (UCLA) activity scores, active range of motion (ROM) and hip–knee–ankle (HKA) angle. The 1‐year postoperative 2011 Knee Society Score, FJS‐12, ROM and HKA angle were recorded.

One‐to‐one matching analysis was performed according to a method described in a previous study [13]. The analysis accounted for age (±5 years), sex (same), body mass index (BMI) (±3 kg/m^2^) and preoperative UCLA activity score (±1 point). Analysis and comparison of the RLLs of the two groups were performed at 2, 4, 6 months and 1 year postoperatively.

### Statistical analysis

Independent Student's *t*‐tests for continuous variables and Fisher's exact test were used to compare the baseline demographics and clinical outcomes. 6‐month and 1‐year postoperative mean maximum widths of RLLs, rates of bone on‐growth and rates of occurrence of RLLs were assessed. All tests were two‐tailed, with statistical significance set to *p* < 0.05. Furthermore, a post hoc power analysis was performed to determine whether the sample size was sufficient. All statistical analyses were carried out in Microsoft R Open 4.0.2 (http://www.r-project.org/).

Based on the sample size of this study, the statistical power was large (1 − *β* = 0.80) across all study parameters to detect significant differences at *α* < 0.05. The ICCs of the intrarater and interrater reliability for the radiological measurements were 0.89 and 0.84, respectively.

## RESULTS

**Figure 3:**
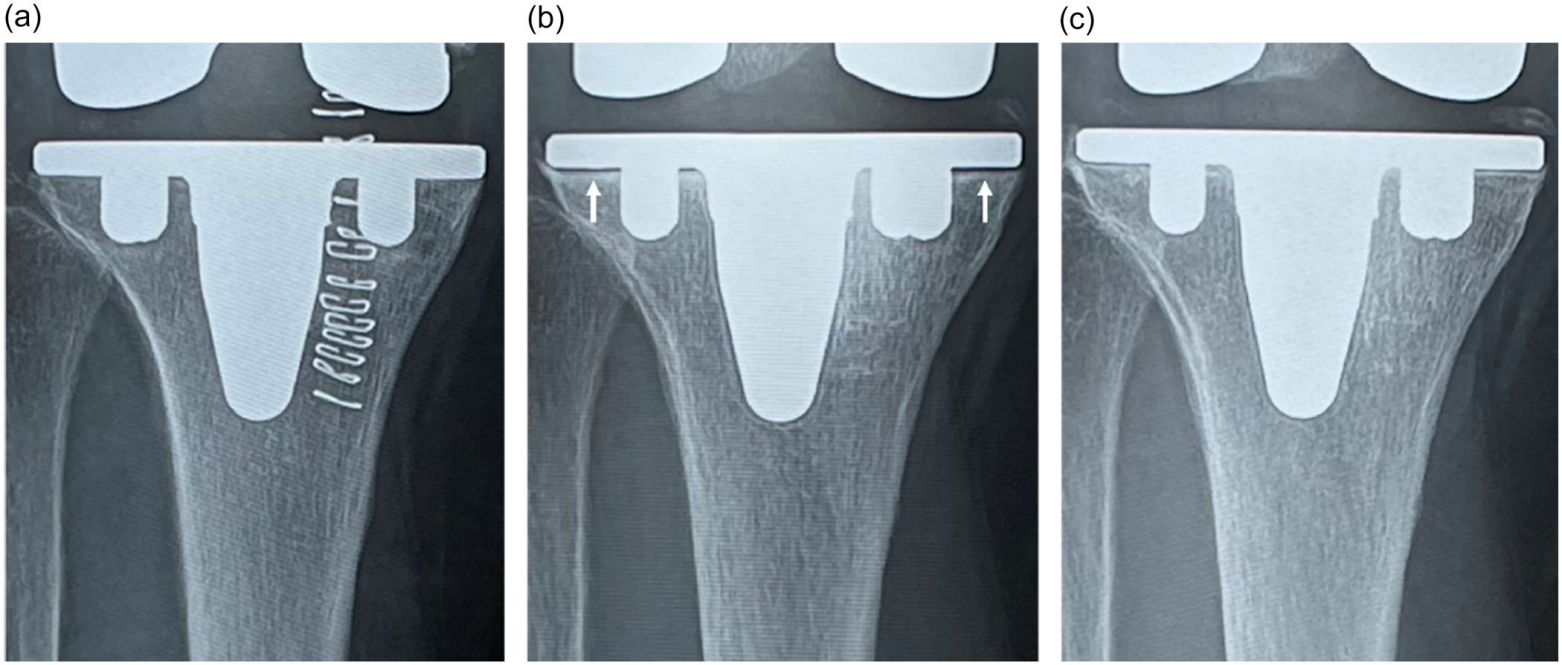
(a) AP radiographs of a patient who underwent POROCOAT TKA at 1 week postoperatively. (b) AP radiographs of a patient who underwent POROCOAT TKA at 6 months postoperatively. RLLs appeared in Zones 1 and 2 (arrow). (c) AP radiographs of a patient who underwent POROCOAT TKA at 1 year postoperatively. RLLs disappeared. AP, anterior–posterior view; RLLs, radiolucent lines; TKA, total knee arthroplasty.

**Figure 4:**
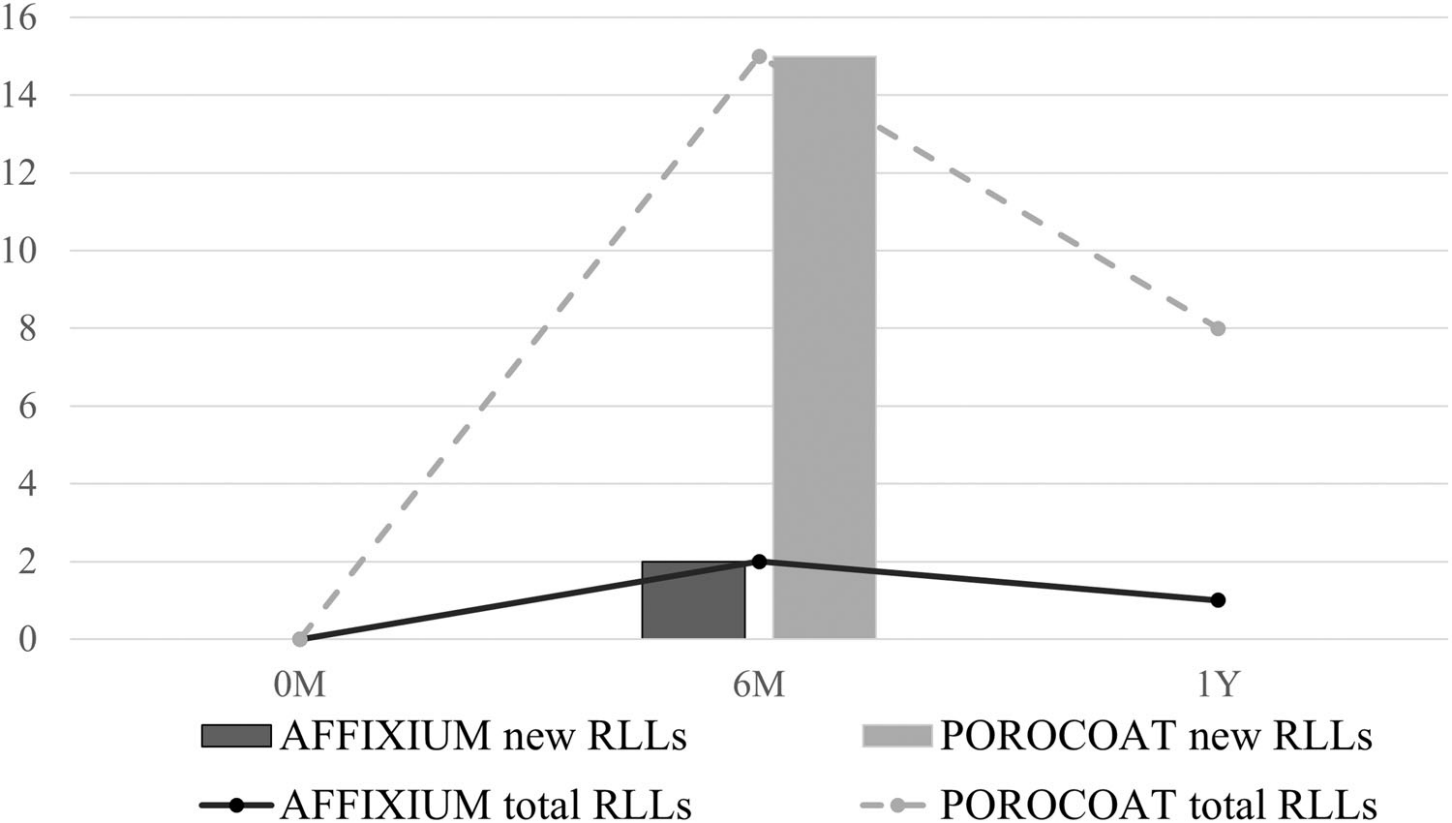
Bar and line graphs show the incidence and total number of cases with RLLs at each time point in the two groups, respectively. RLLs, radiolucent lines.

Fifty AFFIXIUM TKAs and 50 POROCOAT TKAs were successfully matched based on these criteria at the 1‐year follow‐up (Table 1). RLLs appeared in 15 knees (30%) that underwent POROCOAT TKAs at 6 months postoperatively (Figure 3a,b), whereas RLLs appeared in two knees (4%) that underwent AFFIXIUM TKAs (*p *< 0.01). RLLs were predominantly observed in Zones 1 and 2 on AP views of the tibial implant. There was no progression of RLLs in either of the TKAs. RLLs in 6 of 15 POROCOAT TKAs (40%) and 1 of 2 AFFIXIUM TKAs (50%) disappeared 1 year postoperatively (Figures 3c and 4) (Table 2). No knees with RLLs greater than 2 mm were observed, and no subsidence was found during the 1‐year follow‐up in either group.

**Table 2:** Comparison of the 1‐year postoperative radiolucent lines between the matched cohorts.

	POROCOAT *n* width (mm)	AFFIXIUM *n* width (mm)	*p* value
Radiolucent line (total: 6 months)	15	2	<0.01
Tibia, AP (6 months)			
Zone 1	8 (0.68 ± 0.05)	0	
Zone 2	8 (0.68 ± 0.10)	2 (0.84 ± 0.11)	
Tibia, lateral (6 months)			
Zone 1	1 (0.71)	0	
Zone 2	0	0	
Radiolucent line (total: 1 year)	8	1	<0.05
Tibia, AP (1 year)			
Zone 1	5 (0.67 ± 0.10)	0	
Zone 2	4 (0.67 ± 0.12)	10.88	
Tibia, lateral (1 year)			
Zone 1	0	0	
Zone 2	0	0	

Abbreviations: AP, anterior–posterior; *n*, number of knees with radiolucent line; width, mean maximum width of radiolucent line.

**Figure 5:**
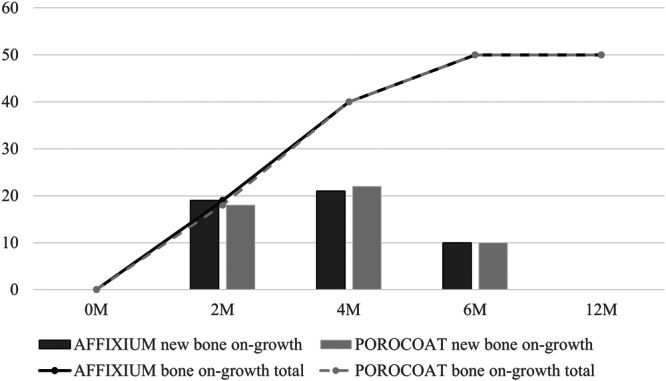
Bar and line graphs show the incidence and total number of cases with bone on‐growth at each time point in the two groups, respectively.

**Figure 6:**
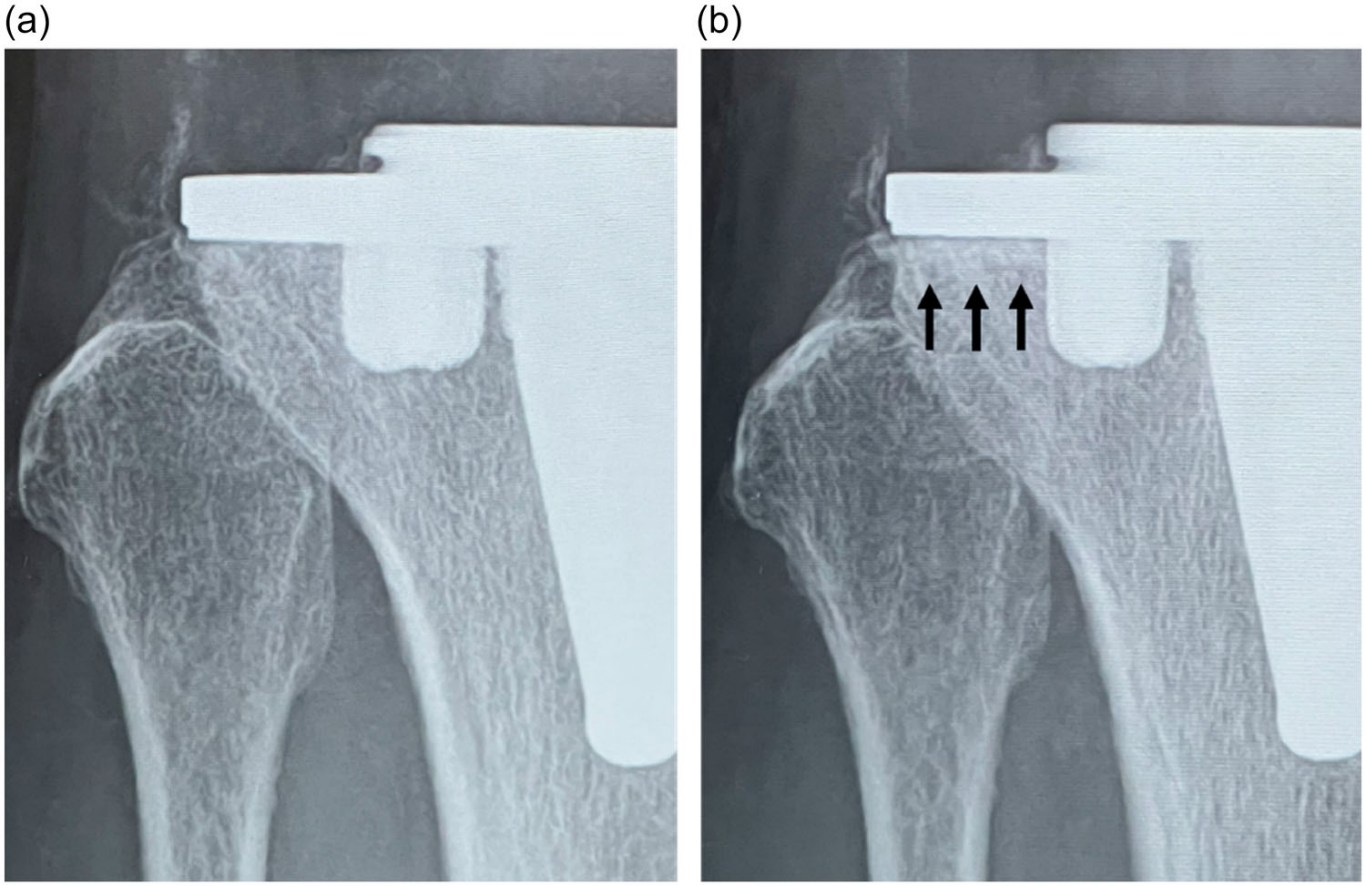
(a) Anteroposterior (AP) radiographs of a patient who underwent AFFIXIUM TKA at 1 week postoperatively. There was no bone on‐growth in Zone 2. (b) AP radiographs of a patient who underwent AFFIXIUM TKA at 2 months postoperatively. Bone on‐growth appeared in Zone 2 (arrow). TKA, total knee arthroplasty.

Bone on‐growth appeared in 18 knees (36%; *p* = 1.00, NS) that underwent POROCOAT TKAs at 2 months postoperatively, 40 knees (80%; *p* = 1.00, NS) at 4 months postoperatively, and 50 knees (100%; *p *= 1.00, NS) at 6 months and 1 year postoperatively (Figure 5) (Table 3). On the other hand, those of AFFIXIUM TKAs were observed in 19 knees (38%; *p *= 1.00, NS) at 2 months postoperatively (Figure 6a,b), 40 knees (80%, *p* = 1.00, NS) at 4 months postoperatively, and 50 knees (100%, *p *= 1.00, NS) at 6 months and 1 year postoperatively (Figure 5) (Table 3).

**Table 3:** Zones and numbers of bone on‐growth over the surface of the implants.

	POROCOAT	AFFIXIUM	*p* value
	*n*	*n*	
Bone on‐growth (total: 2 months)	18	19	1.00 NS
Tibia, AP (2 months)			
Zone 1	7	6	
Zone 2	14	16	
Tibia, lateral (2 months)			
Zone 1	7	7	
Zone 2	0	15	
Bone on‐growth (total: 4 months)	40	40	1.00 NS
Tibia, AP (4 months)			
Zone 1	17	16	
Zone 2	32	26	
Tibia, lateral (4 months)			
Zone 1	14	15	
Zone 2	19	33	
Bone on‐growth (total: 6 months)	50	50	1.00 NS
Tibia, AP (6 months)			
Zone 1	24	20	
Zone 2	45	30	
Tibia, lateral (6 months)			
Zone 1	30	20	
Zone 2	30	42	

Abbreviations: AP, anterior–posterior; *n*, number of knees with bone on‐growth; NS, not significant.

No significant differences were observed in the 2011 Knee Society objective scores, satisfaction scores, expectation scores, functional activity scores, or FJS‐12 scores between the two groups at 1 year postoperatively (Table 4). A significant difference was observed in postoperative ROM (121° ± 11.8° for PROCOAT TKAs and 129.2° ± 8.8° for AFFIXIUM TKAs, *p* < 0.01) (Table 4). No significant difference was observed in postoperative HKA angles between the two groups (*p* = 0.69, NS) (Table 4). None of the TKAs required revision surgery in either group.

**Table 4:** Comparison of postoperative clinical data and radiographic findings between the matched cohorts.

	POROCOAT (*n* = 50)	AFFIXIUM (*n* = 50)	*p* value
2011 Knee Score			
Objective knee score (100 pt)	96.0 ± 13.0	93.8 ± 3.7	0.88 NS
Satisfaction score (40 pt)	31.0 ± 6.2	32.0 ± 4.0	0.53 NS
Expectation score (15 pt)	11.6 ± 5.6	10.0 ± 1.8	0.14 NS
Functional activity score (100 pt)	83.0 ± 11.0	81.1 ± 7.4	0.23 NS
FJS‐12 (pt)	64.5 ± 16.7	61.0 ± 16.0	0.25 NS
HKA angle (d)	179.0 ± 2.3	178.3 ± 2.1	0.10 NS
Postoperative ROM (d)	129.2 ± 8.8	120.0 ± 11.8	**<0.01**

Abbreviations: d, degrees; FJS‐12, Forgotten Joint Score‐12; HKA, hip–knee–ankle; *n*, number of knees; NS, not significant; pt, points; ROM, range of motion.

## DISCUSSION

The most important finding of this study was that RLLs were rarely observed in AFFIXIUM TKAs. This result was different from the common findings of cementless TKAs [13, 14]. Generally, fixed bearings are believed to place more stress on the tibial tray than mobile bearings due to their lower stress dissipation from the tibial tray. Despite its fixed bearing design, a meaningful clinical benefit of AFFIXIUM TKAs over its predecessor was that it did not exhibit a RLL, which may be attributed to the porous material properties of AFFIXIUM.

There are two potential reasons for the significantly lower frequency of RLLs between the bone and tibial baseplate. First, the 3D‐printed titanium porous coating design based on trigonal trapezohedron building blocks is thought to contribute to this result. Three‐dimensional printed titanium porous coatings have high porosity and rougher surfaces, which maximise the initial press‐fit, minimise micromotion and allow for biological fixation [17]. Moreover, Yang et al. reported that less micromotion was observed in AFFIXIUM than in another type of 3D‐printed porous coating in a cadaver study [19]. The porous structure in AFFIXIUM TKAs may have made it easier to purchase into cancellous bone with a high coefficient of friction, resulting in a very low frequency of RLLs.

Excellent initial fixation has also been reported for other types of 3D‐printed porous coatings [5, 16]. Harwin et al. and Sporer et al. reported that Tritanium®, a pure titanium 3D‐printed porous coating, achieved early acquisition of biological fixation [5, 16]. Similarly, Hayakawa et al. reported that Trabecular Metal®, a pure tantalum 3D‐printed porous coating, is structurally similar to cancellous bone with low elasticity, which makes it easier to deflect on the bone contact surface [6]. Tong et al. reported better bony ingrowth in AFFIXIUM® than POROCOAT® in animal models [18]. However, no significant difference was observed in the timing of bone on‐growth between AFFIXIUM TKAs and POROCOAT TKAs in this study. Both designs resulted in excellent bone on‐growth at 6 months postoperatively. Second, the cruciform keel design of the AFFIXIUM TKA may have strengthened the initial fixation. In a saw bone model study, Luo et al. reported that the cementless cruciform keel design demonstrated less micromotion than the central conical keel design [9]. They concluded that the cruciform keel design provided greater stability compared to the conical keel design. However, 100% of knees in both designs achieved good bone on‐growth to the implant on plain radiography, attaining biological fixation at 6 months postoperatively.

Although RLLs were frequently observed in POROCOAT TKAs 6 months postoperatively, 40% of RLLs disappeared within 1 year postoperatively. The cause of RLLs in cementless TKA may be due to micromotions that occur before biological fixation has been achieved [13, 14]. RLLs in cementless TKAs have been observed on both medial and lateral sides in AP radiographs with similar frequency [14]. A similar trend was observed in this study. Furthermore, the vast majority of RLLs in cementless TKA are expected to disappear over time [14]. However, the lack of RLLs after TKA surgery may eliminate some concerns during follow‐up for orthopaedic surgeons who are not familiar with the occurrence of RLLs that are specific to cementless TKA.

The POROCOAT TKAs in this study featured a PS mobile‐bearing design. On the other hand, the AFFIXIUM TKAs featured a PS fixed‐bearing design. There were no significant differences in joint awareness scores or other clinical results except for 1‐year postoperative ROM between the two groups. Amaro et al. and Migliorini et al. conducted meta‐analysis studies and reported that there was no difference between mobile‐bearing and fixed‐bearing TKAs in terms of activities of daily living, pain, joint awareness or survivorship [1, 12]. In this study, the ROM was approximately 8° greater in the mobile‐bearing group. Zinno et al. also reported similar results, as ROM tended to be 7° greater in the Attune PS mobile‐bearing TKA compared to those of a fixed‐bearing TKA [20]. They concluded that the Attune PS mobile‐bearing TKA had greater rotation and translations than the Attune PS fixed‐bearing TKA, resulting in the achievement of greater ROM in their dynamic radiostereometric analysis [20].

This study has several limitations. First, the control group did not include implants with the same bearing design. Thus, the difference in bearing designs may have an impact on the results. This study showed that POROCOAT TKAs had a greater ROM than AFFIXIUM TKAs. The increased ROM may have contributed to the appearance of RLLs. However, it is believed that mobile bearings allow the dissipation of stress from the tibial tray. Comparing two types of coating materials with varying bearings presents a significant challenge, and further studies may be warranted to mitigate the differences between bearing designs. Second, RLLs were only defined as positive when they were detectable in two serial radiographs. However, two serial radiographs were obtained at regular checkups by radiology technicians. Assessment with RLLs provided a simple and clinically relevant means to assess patient follow‐up in cementless TKAs. Third, the assessment was performed by the author (Y.M.) without blinding. However, the ICCs of the intrarater and interrater reliability for them were 0.89 and 0.84, respectively, which were considered to be highly accurate. Lastly, the outcomes of this study are limited to an early to mid‐term follow‐up. Further long‐term studies of 3D‐printed porous‐coated cementless TKA design are needed to validate our results. However, these results are valuable as the lack of progressive RLLs and excellent bone on‐growth over the surface of the implants suggest a successful biological fixation.

## CONCLUSION

A very low frequency of RLLs was observed on a newer 3D‐printed porous‐coated tibial baseplate design for TKA at 1‐year follow‐up. This porous structure may have made it easier to purchase into cancellous bone and strengthen initial fixation.

## AUTHOR CONTRIBUTIONS

Yoshinori Mikashima and Ken Okazaki conceived the study design. Yoshinori Mikashima drafted the manuscript. Katsunori Ikari and Koichiro Yano verified the analytical methods. Ken Okazaki and Hiroshi Takagi supervised the findings of this work. Hitoshi Imamura assisted with the surgeries. All authors discussed the results and contributed to the final manuscript.

## CONFLICT OF INTEREST STATEMENT

Ken Okazaki serves as a consultant for DePuy Synthes and has also received honoraria for lectures from Smith and Nephew. Hiroshi Takagi has also received honoraria for lectures from DePuy Synthes, Smith and Nephew and Zimmer Biomet. Katsunori Ikari serves as a consultant and has received honoraria for lectures from Zimmer Biomet.

## ETHICS STATEMENT

The study was approved by the institutional review board of Oume Knee Surgery Center (Approval Number 2025‐001). The study was performed in accordance with the ethical standards in the 1964 Declaration of Helsinki. All surgeries were performed at the Oume Knee Surgery Center, and the study was conducted at both Oume Knee Surgery Center and Tokyo Women's Medical University. Informed consent was obtained from all patients.

## Supporting information

Affixium: AFFIXIUM® (DePuy Synthes, Warsaw, IN, USA).

Porocoat: POROCOAT® (DePuy Synthes, Warsaw, IN, USA).

## Data Availability

The data that support the findings of this study are not publicly available due to ethical or privacy restrictions. However, they are available from the corresponding author upon reasonable request and with permission from institution and ethics committee.
